# Performance of a Wearable Ring in Controlled Hypoxia: A Prospective Observational Study

**DOI:** 10.2196/54256

**Published:** 2024-06-05

**Authors:** John J Mastrototaro, Michael Leabman, Joe Shumate, Kim L Tompkins

**Affiliations:** 1 Movano Inc dba Movano Health Pleasanton, CA United States

**Keywords:** pulse oximetry, SpO2, pulse oximeter, hypoxia, hypoxemia, clinical trial, accuracy, digital health, wearable, smart ring, ISO 80601-2-61, racial bias

## Abstract

**Background:**

Over recent years, technological advances in wearables have allowed for continuous home monitoring of heart rate and oxygen saturation. These devices have primarily been used for sports and general wellness and may not be suitable for medical decision-making, especially in saturations below 90% and in patients with dark skin color. Wearable clinical-grade saturation of peripheral oxygen (SpO_2_) monitoring can be of great value to patients with chronic diseases, enabling them and their clinicians to better manage their condition with reliable real-time and trend data.

**Objective:**

This study aimed to determine the SpO_2_ accuracy of a wearable ring pulse oximeter compared with arterial oxygen saturation (SaO_2_) in a controlled hypoxia study based on the International Organization for Standardization (ISO) 80601-2-61:2019 standard over the range of 70%-100% SaO_2_ in volunteers with a broad range of skin color (Fitzpatrick I to VI) during nonmotion conditions. In parallel, accuracy was compared with a calibrated clinical-grade reference pulse oximeter (Masimo Radical-7). Acceptable medical device accuracy was defined as a maximum of 4% root mean square error (RMSE) per the ISO 80601-2-61 standard and a maximum of 3.5% RMSE per the US Food and Drug Administration guidance.

**Methods:**

We performed a single-center, blinded hypoxia study of the test device in 11 healthy volunteers at the Hypoxia Research Laboratory, University of California at San Francisco, under the direction of Philip Bickler, MD, PhD, and John Feiner, MD. Each volunteer was connected to a breathing apparatus for the administration of a hypoxic gas mixture. To facilitate frequent blood gas sampling, a radial arterial cannula was placed on either wrist of each participant. One test device was placed on the index finger and another test device was placed on the fingertip. SaO_2_ analysis was performed using an ABL-90 multi-wavelength oximeter.

**Results:**

For the 11 participants included in the analysis, there were 236, 258, and 313 SaO_2_-SpO_2_ data pairs for the test device placed on the finger, the test device placed on the fingertip, and the reference device, respectively. The RMSE of the test device for all participants was 2.1% for either finger or fingertip placement, while the Masimo Radical-7 reference pulse oximeter RMSE was 2.8%, exceeding the standard (4% or less) and the Food and Drug Administration guidance (3.5% or less). Accuracy of SaO_2_-SpO_2_ paired data from the 4 participants with dark skin in the study was separately analyzed for both test device placements and the reference device. The test and reference devices exceeded the minimum accuracy requirements for a medical device with RMSE at 1.8% (finger) and 1.6% (fingertip) and for the reference device at 2.9%.

**Conclusions:**

The wearable ring meets an acceptable standard of accuracy for clinical-grade SpO_2_ under nonmotion conditions without regard to skin color.

**Trial Registration:**

ClinicalTrials.gov NCT05920278; https://clinicaltrials.gov/study/NCT05920278

## Introduction

The ability to seamlessly monitor health metrics at home with a wearable device that meets accuracy standards as provided by the US Food and Drug Administration (FDA) [1] may have great value in the care of individuals living with chronic conditions, as well as in preventative care. Providing health monitoring at home has many possible benefits, including the potential to reduce the costs of health care, provide care to people without access to clinical resources, identify potential health concerns early so care can be delivered in a timely fashion, and help individuals take more active control of their health, empowering them with a better understanding of how their lifestyle affects their health metrics.

Wearable devices to monitor health are available in several form factors, including patches, watches, rings, and more recently, wearable biosensors including gloves and shirts. Wearables can offer clinical monitoring of metrics including peripheral blood oxygen saturation, glucose, and blood pressure. Wearables are available in consumer grade, which is suitable for fitness use, and also in clinical grade, which is suitable for medical monitoring typically under the direction and care of a physician or other health care provider.

The test device aims to address health monitoring needs through a smart ring pulse oximeter which will monitor clinical-grade SpO_2_ and pulse rate at rest but also provide wellness metrics to provide a broad overview of health. The wellness metrics that the test ring provides include heart rate variability, average SpO_2_, respiration rate, sleep duration and stages, and skin temperature during sleep, in addition to skin activity levels (steps), distance traveled, active minutes, and calories burned when awake, and also enables tracking of mood and menstrual cycle.

A pulse oximeter provides SpO_2_, a noninvasive estimate of arterial blood oxygen saturation (SaO_2_). Spot checks at home using pulse oximeters are important in detecting low oxygen levels that require medical intervention and can play an important role in understanding health trends, especially when dealing with chronic conditions such as chronic obstructive pulmonary disease, congestive heart failure, asthma, bronchitis, anemia, cystic fibrosis, and sleep apnea. In addition, SpO_2_ monitoring has been important in understanding the severity of acute respiratory illness including COVID-19 [2] on the pulmonary system. Essentially, any condition affecting breathing or oxygen uptake would benefit from SpO_2_ monitoring provided the data meets accuracy standards.

SpO_2_ accuracy can be affected by sensor technology and placement, and by certain patient characteristics. For example, it has been known since 1990 [3] that skin color may affect measured SpO_2_, with pulse oximeters typically overestimating SaO_2_ in dark skin in the presence of occult hypoxemia [4-6]. Skin color is impacted not only by melanin pigment but also by blood flow, skin thickness, and photoaging [7]. Other patient factors that can affect noninvasive pulse oximeter accuracy with optical sensors are anemia, skin temperature, tobacco use, and fingernail polish [8]. These known causes of SpO_2_ overestimation inaccuracies for patients with dark skin color may result in significant underdiagnosis and undertreatment, ultimately contributing to poor health outcomes and additional health disparities [9]. Accuracy and racial bias vary by device and manufacturer with clinical-grade medical devices meeting FDA requirements for commercialization in the United States [10,11].

In its guidance document, the FDA has recognized the International Organization for Standardization (ISO) 80601-2-61:2019 [12] for the evaluation of the safety and effectiveness of pulse oximeter devices, and the clinical study reported here used a methodology consistent with the standard. The two important objectives of the defined clinical study requirements in the standard are: (1) to evaluate the sensors over a broad range of SpO_2_ values (70%-100%) when used per device instructions, and (2) to ensure the device provides accurate data across the range of skin colors including dark skin.

## Methods

### Overview

**Figure 1:**
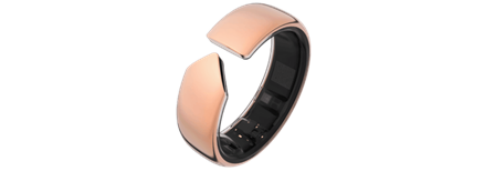
Image of the ring test device. The pulse oximeter sensors can be seen on the inner surface of the ring, which will fit against the palmar side of a finger.

The test device is a rechargeable, battery-operated ring design with working components including a printed circuit board assembly, battery, and sensors placed between a metal outer shell and a plastic inner ring (Figure 1). The test device uses reflectance photoplethysmography technology of 2 optical sensors with 2 light-emitting diodes or transmitters of multi-wavelengths (526-940 nm) and a photodiode receiver on the same side of a cutaneous vascular bed [13]. The reflected light absorption of oxygenated and deoxygenated blood is captured and the oxygen saturation ratio is calculated to noninvasively monitor in vivo SpO_2_. The ring collects, processes, and stores data, and then sends data wirelessly to a compatible and connected smartphone where data are further processed, displayed, and stored using companion application software. For the study, test device data were continuously collected.

### Study Design

The study was an open enrollment, single-center, single-blinded design using the investigational ring compared with arterial blood gas measurements (SaO_2_) and 2 commercially available hospital grade reference pulse oximeters (Massimo Radical-7 [14] and Nellcor N-595 [15]). The study was conducted at an independent lab (Hypoxia Research Laboratory, Department of Anesthesia and Perioperative Care, University of California San Francisco) under the direction of Phillip Bickler, MD, PhD, and John Feiner, MD, and sponsored by Movano Health (Pleasanton, California). The clinical study reported here used a methodology for accuracy consistent with the FDA guidance document [10].

### Ethical Considerations

The test device, study, and study consent were reviewed and approved for use in a nonsignificant-risk clinical trial by the University of California San Francisco Committee on Human Research (ID 379826). Written informed consent was obtained from all participants prior to testing. Participants were paid US $200 for their participation. All data used in the analysis were deidentified prior to analysis.

### Inclusion and Exclusion Criteria

To be included in the study, participants had to meet all the inclusion criteria and not meet any of the exclusion criteria. For inclusion, participants had to be in general good health, between 18 and 50 years old, fluent in written and spoken English, willing to provide consent and comply with study procedures, and willing to have their skin color assessed with a commercial instrument designed for that purpose. Participants were excluded from participation if they did not meet the inclusion criteria or if they were obese (defined as a BMI of 30 or greater), a current smoker, diabetic, pregnant, lactating, or trying to get pregnant; had a known diagnosis of Raynaud disease, asthma, sleep apnea (or were currently using a continuous positive airway pressure device), clotting disorder, hemoglobinopathy or history of anemia, or liver, heart, lung, kidney disease or any other serious systemic disease; had a history of fainting or vasovagal response or sensitivity to anesthesia; or if, in the opinion of the investigator, they were unsuitable for participation for any reason or based on the first blood draw, the participant had an unacceptable Allen test [16], the participant had any systemic serious illness, the participant had any injury, deformity, or abnormality at the sensor sites that would interfere with the sensors working properly, or any other condition that would make them an unsuitable participant.

### Procedure for Desaturation

Demographic data were recorded immediately following written consent including self-reported ethnicity. In addition, trained clinical research staff evaluated the skin color of each participant using the Fitzpatrick scale, the Monk scale, and a spectrometer for evaluation and potential future use [17]. Participant skin pigmentation was assessed at 5 different skin locations of each participant including the dorsal surface of the finger between the nail bed and the distal interphalangeal joint, the palmar surface of the distal phalanx, the inner upper arm, the front and back of the earlobe, and the external nare. The skin color of the dorsal distal index finger using the Fitzpatrick scale was used for skin color determination. Independently, participants were asked to self-identify their ethnicity, and facial skin tone on the forehead was measured using a spectrometer to ensure the optical monitoring approach was accurate across different ethnicities and skin colors. Participants in this study had various levels of skin color, ranging from very fair to dark or I to VI using the Monk scale and the Fitzpatrick scale, respectively [18-20].

Each participant had 2 test devices placed: 1 at the base of the finger and 1 at the distal tip, similar to most commercially available pulse oximeters. In addition, calibrated, hospital-grade co-oximeters (Massimo Radical-7 and Nellcor N-595) were placed on the distal tips of fingers and used as reference devices. A radial arterial cannula was placed in either the left or right wrist of each participant for arterial blood sampling and blood pressure monitoring. Blood gas analysis, the gold standard to determine oxyhemoglobin saturation (SaO_2_), was performed using an ABL-90 multi-wavelength oximeter [21] (Hemoximeter, Radiometer, Copenhagen, serial 1393-090R0359N0002). This instrument is factory calibrated and contains quality control algorithms.

**Figure 2:**
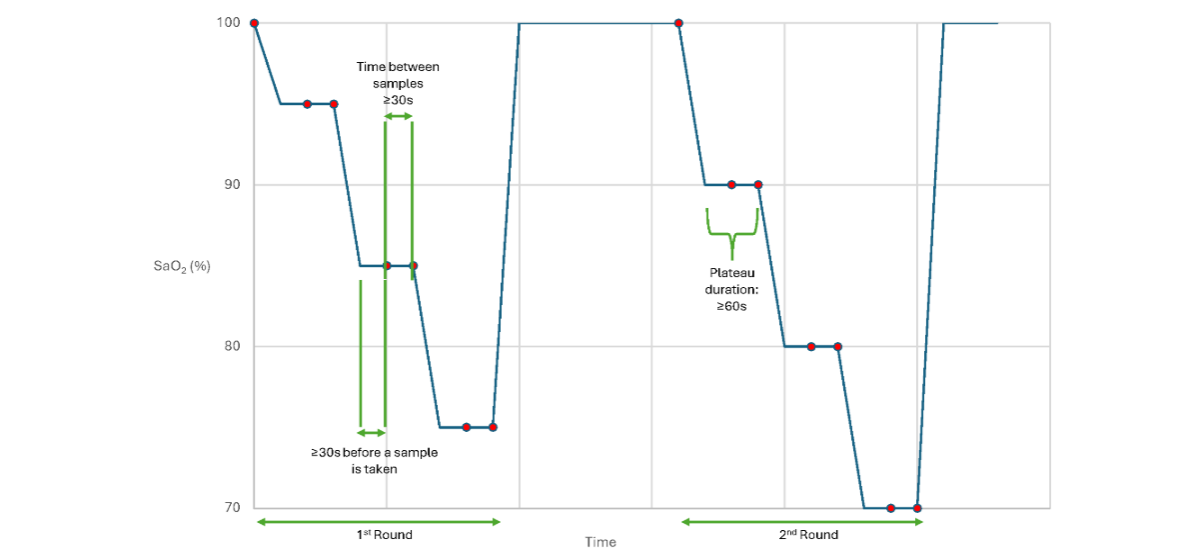
Target plateaus of oxygen saturation and sampling points. The blue dots represent the SaO2 value at each sampling point. According to the SaO2 stability standard of the pulse oximeter probe placement site, the first sample at each plateau was acquired 30 seconds after reaching a target plateau, and the successive sample was acquired at least 30 seconds after the first sample.

Each participant had 2 control blood samples taken at the beginning of each experiment while breathing room air. Hands with test devices and reference pulse oximeters were maintained motionless on arm boards throughout the test. Hypoxemia was then induced to different and stable levels of oxyhemoglobin saturation (between 70% and 100%) by having participants breathe mixtures of nitrogen, air, and carbon dioxide. The mixture of gases was controlled by the study physician by adjusting gas flows according to breath-by-breath estimates of oxygen saturation calculated from end-tidal partial pressure of oxygen (PO_2_) and partial pressure of carbon dioxide (PCO_2_) displayed on a screen using LabVIEW (National Instruments) software 2015 [22,23]. Each plateau level of oxyhemoglobin saturation was maintained for at least 30 seconds or until readings of the reference pulse oximeters were stable. A total of 2 arterial blood samples were then obtained, approximately 30 seconds apart. Each stable plateau was maintained for at least 60 seconds with SpO_2_ fluctuating by less than 2%-3%. Target plateaus and arterial blood gas sampling points are shown in Figure 2.

Overall, at least 230 arterial blood gas measurements were obtained and at least 200 paired data points were collected for each investigational device placement and reference device combination studied. After the study, data from the test devices during arterial blood sampling times were provided to the clinical site for independent analysis (Multimedia Appendix 1).

### Data Analysis

Test device data were taken at 5-second averages corresponding to the time when arterial blood samples were obtained. Individual data points were missed or excluded for dropped signals or failure of the oximeter signal to achieve an appropriate plateau. Participant 2 was used to calibrate the test data and was not included in the analysis.

Data were plotted as hemoximeter data (SaO_2_) compared with each test device and Masimo Radical-7 reference pulse oximeter (SpO_2_) data with bias calculated as SpO_2_–SaO_2_. Linear regression was plotted for all 11 participants’ studies combined, and the equation *R*^2^ was calculated. Mean bias and the upper and lower limits of agreement (mean bias, SD 1.96) were calculated and tabulated. Limits of agreement were adjusted when there were multiple readings per participant and multiple participants according to the modified Bland and Altman methodology [24]. For the pooled plots, different markers were used for each participant.

Tables of count, missing data, mean, SD, SE, minimum, maximum, 95% CI, and accuracy root mean square error (RMSE) were provided for each oximeter’s bias, and all oximeters combined in the following ranges of SaO_2_ (hemoximeter) deciles: 60%-70%, 70%-80%, 80%-90%, and 90%-100%, and over the test range 70%-100%. A table of mean bias and RMSE was provided for each oximeter. A table of variances within and between study participants was provided for each oximeter.

Accuracy root mean square or RMSE was calculated as follows:



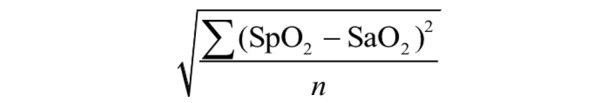



## Results

This study was performed on October 26 and 27, 2022, and included 11 participants consisting of 5 women and 6 men in the accuracy analysis. All the participants enrolled in the study met the inclusion and exclusion criteria, had hemoglobin levels ≥10 gm/dl, and were healthy nonsmoking individuals aged 22-34 years. A distribution of ethnicities and skin color based on the Monk scale [25] and the Fitzpatrick scale were studied, with 4 participants having dark skin color both using a modified Monk scale (light, medium, and dark) and the Fitzpatrick scale type V or VI, with 3 of the 4 self-reported as African American. The demographic data of the participants are provided in Table 1.

The baseline and desaturation plateaus were nominally at room air and 100%, 93%, 90%, 87%, 85%, 82%, 80%, 77%, 75%, and 70% saturation. No adverse events occurred during the study. A total of 258 samples were obtained for the test device on the fingertip and 236 samples were obtained for the test device on the finger over the saturation plateaus. Tables present the statistical results for both the finger placement (Table 2) and the fingertip placement (Table 3) of the test device. The Masimo [14] Radical-7 [26] was selected as a reference pulse oximeter for comparison and device statistical results (Table 4) were included. The overall RMSE for the test device was approximately 2.1% for both the finger placement (2.07%) and the fingertip placement (2.11%) compared with SaO_2_ while the overall RMSE for the Masimo Radical-7 reference device was 2.8% compared with SaO_2_.

**Figure 3:**
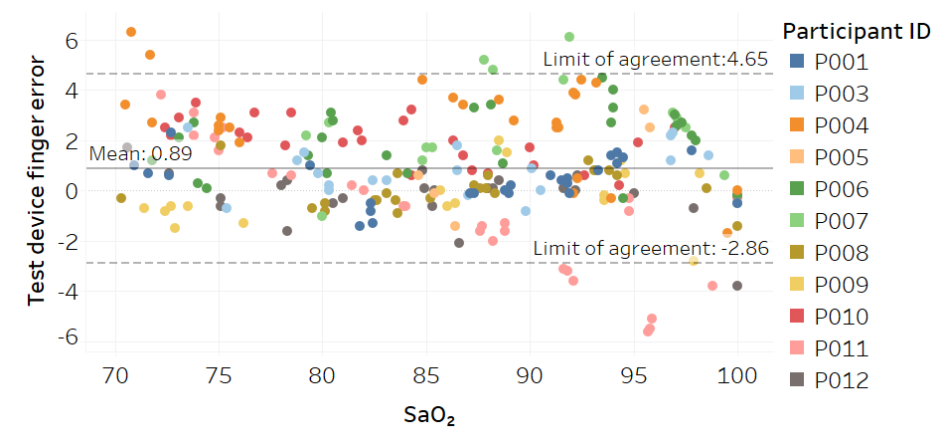
Modified Bland-Altman plot for the test device with finger placement.

**Figure 4:**
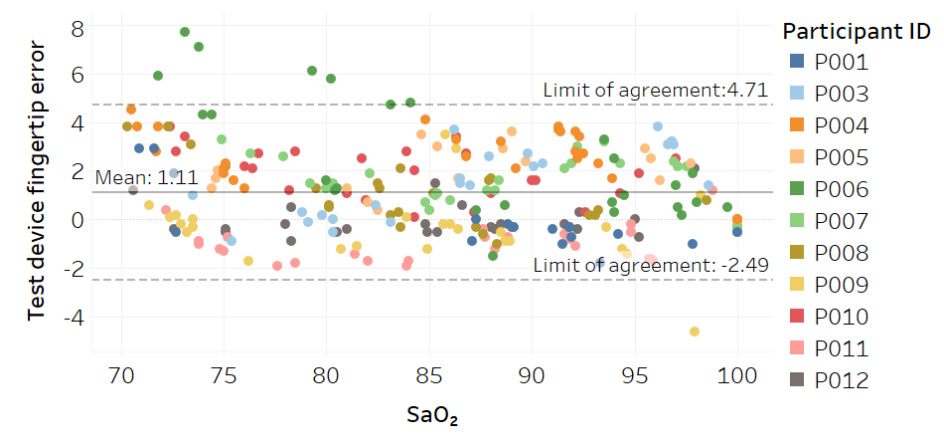
Modified Bland-Altman plot for the test device with fingertip placement.

**Figure 5:**
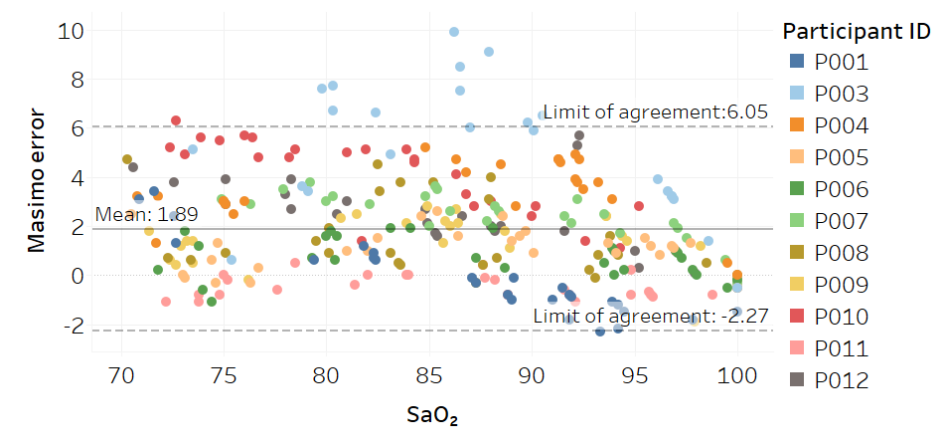
Modified Bland-Altman plot for the Masimo Radical-7 reference device.

**Figure 6:**
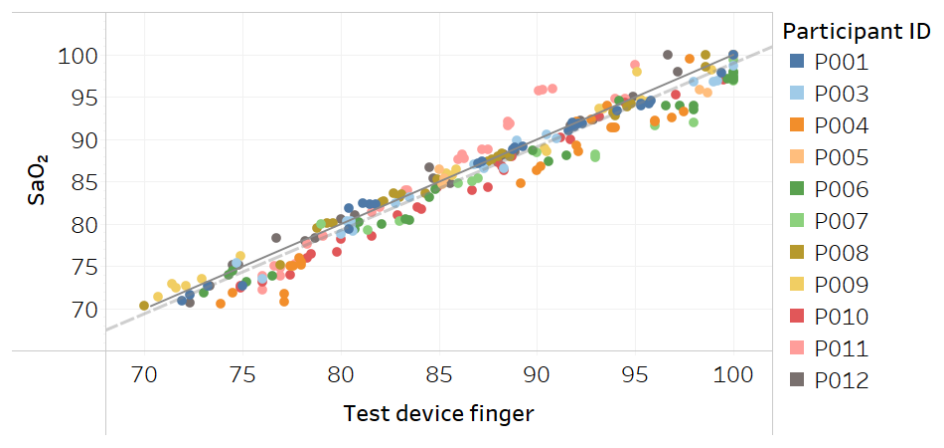
Correlation plot of SaO_2_ versus test device finger placement SpO_2_ for all participants. The solid line is a diagonal reference line. The dotted line is the linear regression line of fit with the formula SaO_2_=0.988 × test device finger + 0.188, R_2_=0.95.

**Figure 7:**
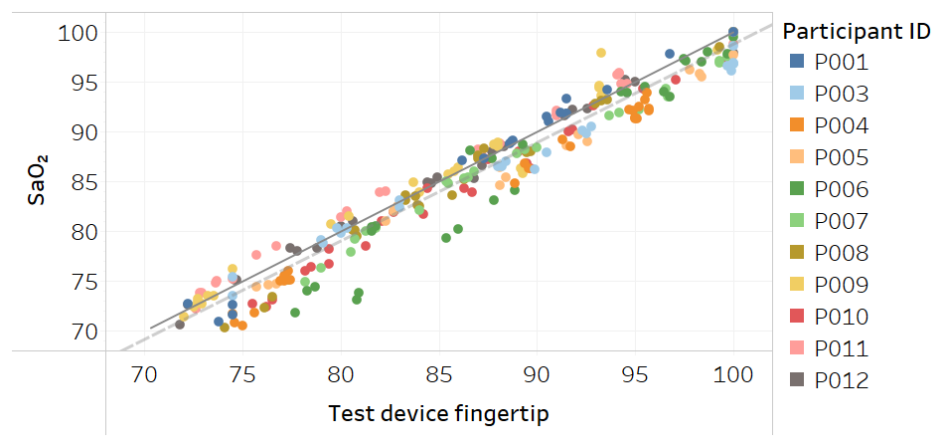
Correlation plot of SaO_2_ versus test device fingertip placement SpO_2_ for all participants. The solid line is a diagonal reference line. The dotted line is the linear regression line of fit with the formula SaO_2_=0.990 × test device fingertip – 0.199, R_2_=0.95.

**Figure 8:**
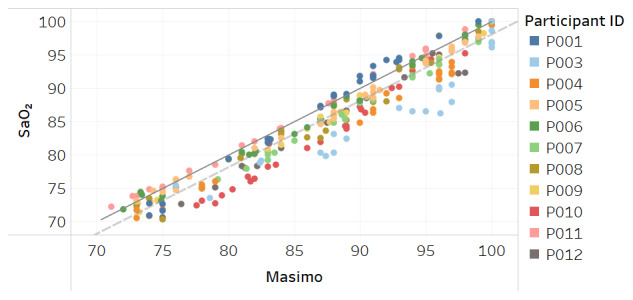
Correlation plot of SaO_2_ versus Masimo Radical-7 reference device SpO_2_ for all participants. The solid line is a diagonal reference line. The dotted line is the linear regression line of fit with the formula SaO_2_=0.997 × Masimo – 1.682, R_2_=0.94.

Modified Bland-Altman plots of the test devices by placement (finger and fingertip) and of the Masimo co-oximeter reference device are shown in Figures 3-5. Correlation plots with linear regression lines of best fit for all participants for the 3 devices are presented in Figures 6-8.

**Figure 9:**
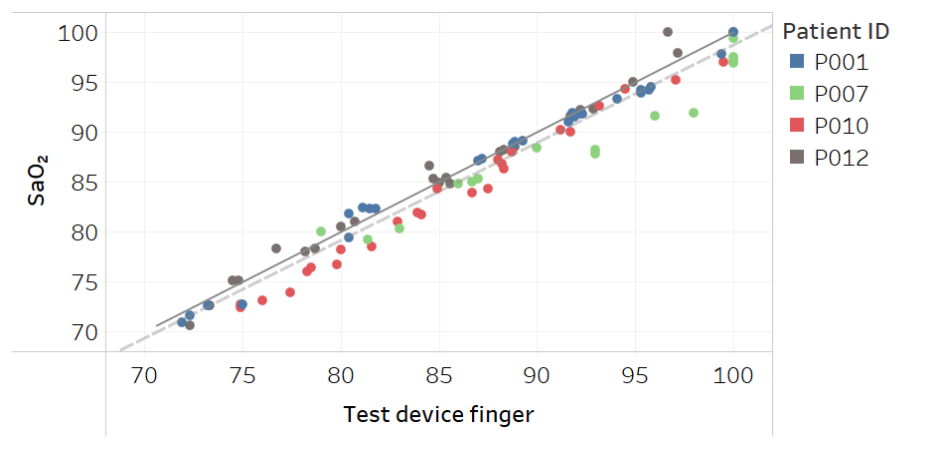
Correlation plot of SaO_2_ versus test device finger placement SpO_2_ for participants determined to be V-VI or dark-skinned using the Fitzpatrick scale. The solid line is a diagonal reference line. The dotted line is the linear regression line of fit with the formula SaO_2_=0.981 × test device finger + 0.577, R_2_=0.96.

**Figure 10:**
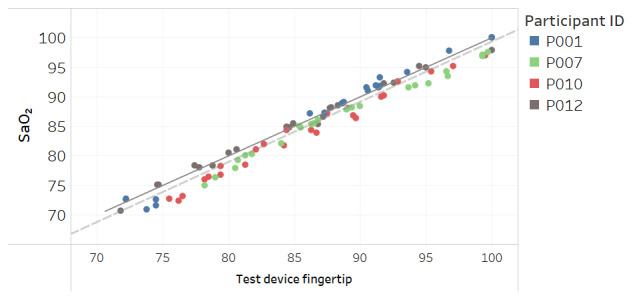
Correlation plot of SaO_2_ versus test device fingertip placement SpO_2_ for participants determined to be V-VI or dark-skinned using the Fitzpatrick scale. The solid line is a diagonal reference line. The dotted line is the linear regression line of fit with the formula SaO_2_=1.022 × test device fingertip – 2.849, R_2_=0.95.

**Figure 11:**
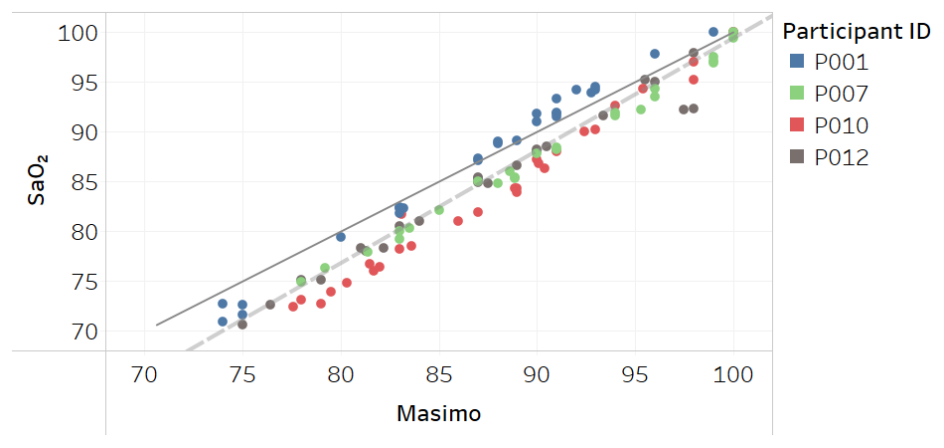
Correlation plot of SaO_2_ versus Masimo Radical-7 reference device SpO_2_ for participants determined to be V-VI or dark-skinned using the Fitzpatrick scale. The solid line is a diagonal reference line. The dotted line is the linear regression line of fit with the formula SaO_2_=1.133 × Masimo – 13.878, R_2_=0.95.

We performed a secondary analysis to explore whether the devices accurately measured SpO_2_ in dark skin due to concerns that have been raised with traditional pulse oximeters before and throughout COVID-19 [27] where it was shown the oximeters may overread the actual oxygen concentration in dark skin [6]. In this secondary analysis, we included the Masimo Radical-7 reference device. Figures 9-11 show the results solely from participants with dark skin using the Fitzpatrick scale V and VI participants. As is shown, the test device maintains strong accuracy over the range of 70%-100% with an RMSE of 1.8% for the traditional ring or finger placement, and 1.6% for fingertip placement, whereas the Masimo Radical-7 reference oximeter RMSE was 2.9% with a bias of 2.2% over the same range. Accuracy degrades for the Masimo Radical-7 device at lower SaO_2_ measurements with a bias to overread SpO_2_ compared with SaO_2_ in dark skin.

**Table 1:** Demographic data.

Participant number	Gender	Age (years)	BMI	Ethnicity	Monk scale	Fitzpatrick scale
1	Man	26	19	African American	9/10 (dark)	Type V
3	Woman	26	22	Caucasian	5/6 (medium)	Type III
4	Man	34	21	Caucasian	1/2 (light)	Type I
5	Woman	25	29	Caucasian	1/2 (light)	Type II
6	Woman	22	22	Multiethnic	5/6 (medium)	Type IV
7	Man	28	20	Asian	9/10 (dark)	Type V
8	Man	25	24	Asian	5/6 (medium)	Type IV
9	Woman	24	21	Asian	5/6 (medium)	Type III
10	Man	33	24	African American	9/10 (dark)	Type VI
11	Woman	22	18	Multiethnic	5/6 (medium)	Type IV
12	Man	31	24	African American	9/10 (dark)	Type VI

**Table 2:** Test device bias for finger placement.

	Hemoximeter range				
	60-70	70-80	80-90	90-100	70-100
Count of data, n	9	59	90	87	236
Count of missing data, n	0	23	28	27	78
Mean (SD) (%)	2.48 (2.04)	1.42 (1.64)	0.66 (1.58)	0.78 (2.22)	0.89 (1.88)
SE (%)	0.68	0.21	0.17	0.24	0.12
95% confidence limit (%)	1.56	0.43	0.33	0.47	0.24
Limits of agreement (%)	N/A^a^	–1.90, 4.75	–2.55, 3.86	–3.70, 5.25	–2.86, 4.65
Maximum (%)	5.30	6.30	5.20	6.10	6.30
Minimum (%)	–0.40	–1.60	–2.10	–5.60	–5.60
Root mean square error (%)	3.13	2.16	1.71	2.34	2.07

^a^N/A: not applicable.

**Table 3:** Test device bias for fingertip placement.

	Hemoximeter range				
	60-70	70-80	80-90	90-100	70-100
Count of data, n	9	66	104	88	258
Count of missing data, n	0	16	14	26	56
Mean (SD) (%)	3.31 (1.45)	1.62 (2.15)	0.89 (1.60)	0.98 (1.66)	1.11 (1.80)
SE (%)	0.48	0.27	0.16	0.18	0.11
95% confidence limit (%)	1.11	0.53	0.31	0.35	0.22
Limits of agreement (%)	N/A^a^	–2.76, 6.01	–2.31, 4.09	–2.37, 4.34	–2.49, 4.71
Maximum (%)	5.20	7.70	5.80	3.80	7.70
Minimum (%)	0.96	–1.90	–1.90	–4.60	–4.60
Root mean square error (%)	3.58	2.68	1.82	1.92	2.11

^a^N/A: not applicable.

**Table 4:** Masimo Radical-7 device bias.

	Hemoximeter range				
	60-70	70-80	80-90	90-100	70-100
Count of data, n	9	82	117	114	313
Count of missing data, n	0	0	1	0	1
Mean (SD) (%)	3.90 (1.57)	2.09 (2.06)	2.41 (2.12)	1.21 (1.85)	1.89 (2.07)
SE (%)	0.52	0.23	0.20	0.17	0.12
95% confidence limit (%)	1.21	0.45	0.39	0.34	0.23
Limits of agreement (%)	0.84, 6.96	–2.07, 6.26	–1.89, 6.70	–2.51, 4.93	–2.27, 6.05
Maximum (%)	6.60	7.60	9.90	6.50	9.90
Minimum (%)	2.10	–1.10	–1.00	–2.30	–2.30
Root mean square error (%)	4.17	2.93	3.20	2.20	2.80

## Discussion

### Principal Findings

Over the past several years as people have become more aware and attuned to their health, especially through the course of the COVID-19 pandemic, wearable products have expanded in popularity and matured to provide additional features including the monitoring of pulse and blood oxygen saturation. Although most wearables are wellness devices, there is a need for clinical-grade wearables that may have utility in both the clinical management of chronic conditions, as well as prevention.

The test device is designed to serve this purpose and has demonstrated clinical-grade accuracy in the monitoring of peripheral oxygenation saturation or SpO_2_. SpO_2_ and pulse are essential elements of the product solution, although monitoring of activity, sleep, skin surface temperature, and other metrics helps to provide a more comprehensive picture of health.

This study tested the investigational ring as a clinical-grade pulse oximeter both when worn at the base of a finger and also when held at the fingertip. Collected noninvasive investigational SpO_2_ data were then compared with invasive SaO_2_ arterial blood gas measurements and other commercially available, hospital-grade devices, according to an internationally accepted standard for demonstrating accuracy.

Acceptable maximum RMSE accuracy for medical devices per the international standard is 4% and per FDA is 3.5%. Over the SpO_2_ range, the test device RMSE was 2.1% when worn on the finger at the base and also when held between the thumb and fingertip. While this compared favorably to RMSE of 2.8% for the Masimo Radical-7 reference device in the study, all devices tested met the minimum accuracy requirements. The correlation plots and associated linear regression formulas show a strong relationship between estimates and actual SaO_2_ values, evidenced by slope terms being near 1 and intercept values being close to 0. The one departure from this trend is Figure 11 for the Masimo device in dark skin, where the regression line is not parallel to the diagonal reference line, indicating differing levels of accuracy between low and high values of SaO_2_. The intercept term of –13.9 is also an indication of a lack of fit.

### Limitations

There are several limitations of this study. First, in accordance with the standard and FDA guidance, the study is based on a small sample of healthy, young adult volunteers and may not apply to younger, older, more diverse, or ill patients. Due to the nature of the study, the standard requires a total of 10 participants, with at least 20% of the samples from participants with dark skin. Accuracy in this study was determined using 11 participants, with 36% of participants with dark skin.

Another limitation is the use of the Fitzpatrick and Monk scales to determine skin color. These tools are flawed as they are subjective and not developed for this purpose. Better tools are being tested including spectrometers [17]. Once validated and readily available, spectrometers designed to measure skin color should help remove subjectivity and ensure consistency across studies and in clinical practice.

### Conclusion

The results of this study demonstrated the ability of the test device to accurately monitor SpO_2_ as a ring and a fingertip medical device, meeting the FDA and ISO standard accuracy requirements of RMSE less than 3.5% and 4%, respectively. Furthermore, the test devices met an RMSE of less than 3% for all participants for both the finger and fingertip placements of the test device (RMSE 2.1%) as proposed by Okunlola et al [28] and for the subset of participants with dark skin color (4/11, 36%) in the study (RMSE 1.8% finger and 1.6%). By any of these standards, the test data supports accuracy for a clinical-grade pulse oximeter medical device over the fully tested range of 70% to 100% SaO_2_ when worn on a finger or held between the fingertip and thumb for all participants and participants with dark skin. This bodes well for the future of FDA-cleared home wearables with optimized reflective optical technology providing accurate data for the user and their clinician alike.

## Appendix

Multimedia Appendix 1 Test data.

## Abbreviations

FDA: Food and Drug Administration
ISO: International Organization for Standardization
RMSE: root mean square error
