# Supplementary material for: Performance of a Wearable Ring in Controlled Hypoxia: A Prospective Observational Study
Source: JMIR Form Res. 2024 Jun 5;8:e54256. doi: 10.2196/54256 (PMC11187508; doi:10.2196/54256)
Supplement: Multimedia Appendix 1 [file formative_v8i1e54256_app1.docx]

Appendix 1: Test Data

| **Subject** | **Sample** | **SaO2**  **ABL 90**  **(%)** | **SpO2**  **Test fingertip (%)** | **SpO2**  **Test finger (%)** | **SpO2 Masimo (%)** | **SpO2 Nellcor (%)** |
| --- | --- | --- | --- | --- | --- | --- |
| Subject #1 | 1 | 97.8 | 96.8 | 99.4 | 96.0 | 97.0 |
| Subject #1 | 2 | 94.2 | 93.6 | 95.3 | 92.0 | 93.0 |
| Subject #1 | 3 | 94.5 |  | 95.8 | 93.0 | 93.1 |
| Subject #1 | 4 | 94.2 |  | 95.7 | 93.0 | 93.0 |
| Subject #1 | 5 | 93.9 |  | 95.3 | 92.8 | 93.0 |
| Subject #1 | 6 | 93.3 | 91.5 | 94.1 | 91.0 | 92.0 |
| Subject #1 | 7 | 91.8 | 91.5 | 92.3 | 90.0 | 91.0 |
| Subject #1 | 8 | 91.8 | 91.5 | 92.1 | 91.0 | 91.0 |
| Subject #1 | 9 | 91.9 | 91.2 | 91.8 | 91.0 | 91.0 |
| Subject #1 | 10 | 91.5 | 90.5 | 91.9 | 91.0 | 91.0 |
| Subject #1 | 11 | 91.0 | 90.6 | 91.6 | 90.0 | 90.2 |
| Subject #1 | 12 | 89.1 | 88.8 | 89.3 | 89.0 | 89.0 |
| Subject #1 | 13 | 89.0 | 88.7 | 88.9 | 88.0 | 89.0 |
| Subject #1 | 14 | 88.8 | 88.6 | 88.8 | 88.0 | 89.0 |
| Subject #1 | 15 | 87.3 | 87.3 | 87.2 | 87.0 | 87.0 |
| Subject #1 | 16 | 87.1 | 86.2 | 87.0 | 87.0 | 88.0 |
| Subject #1 | 17 | 79.4 |  | 80.4 | 80.0 | 81.0 |
| Subject #1 | 18 | 82.3 |  | 81.8 | 83.2 | 84.0 |
| Subject #1 | 19 | 82.3 |  | 81.5 | 83.0 | 83.0 |
| Subject #1 | 20 | 82.4 |  | 81.1 | 83.0 | 82.7 |
| Subject #1 | 21 | 81.8 |  | 80.4 | 83.0 | 82.0 |
| Subject #1 | 22 | 72.6 | 74.5 | 73.2 | 75.0 | 75.0 |
| Subject #1 | 23 | 72.7 |  | 72.2 | 75.0 | 74.0 |
| Subject #1 | 24 | 69.9 | 72.9 | 70.1 | 72.0 | 72.0 |
| Subject #1 | 25 | 71.6 | 74.5 | 72.3 | 75.0 | 74.0 |
| Subject #1 | 26 | 70.9 | 73.8 | 71.9 | 74.0 | 71.9 |
| Subject #1 | 27 | 100.5 | 100.0 | 100.0 | 99.0 | 100.0 |
| Subject #3 | 1 | 98.6 | 100.0 | 100.0 | 100.0 | 100.0 |
| Subject #3 | 2 | 96.9 | 100.0 | 99.2 | 100.0 | 98.0 |
| Subject #3 | 3 | 96.8 | 100.0 | 99.0 | 100.0 | 98.0 |
| Subject #3 | 4 | 96.8 | 100.0 | 98.0 | 100.0 | 98.0 |
| Subject #3 | 5 | 89.8 | 92.5 | 89.0 | 96.0 | 92.0 |
| Subject #3 | 6 | 90.1 | 92.3 | 91.0 | 96.0 | 92.0 |
| Subject #3 | 7 | 90.5 | 92.8 | 90.5 | 97.0 | 93.0 |
| Subject #3 | 8 | 87.0 | 88.4 | 86.8 | 93.0 | 89.0 |
| Subject #3 | 9 | 86.5 | 88.2 | 87.3 | 94.0 | 89.0 |
| Subject #3 | 10 | 86.5 | 88.0 | 88.3 | 95.0 | 89.0 |
| Subject #3 | 11 | 80.3 | 80.3 | 80.3 | 87.0 | 82.2 |
| Subject #3 | 12 | 80.3 | 79.8 | 80.5 | 88.0 | 82.0 |
| Subject #3 | 13 | 79.8 | 80.0 | 80.5 | 87.4 | 83.0 |
| Subject #3 | 14 | 68.2 | 71.8 | 73.5 | 74.8 | 68.0 |
| Subject #3 | 15 | 67.4 | 72.0 | 72.7 | 73.0 | 67.0 |
| Subject #3 | 16 | 68.8 | 72.0 | 71.9 | 71.0 | 67.1 |
| Subject #3 | 17 | 100.5 | 100.0 | 100.0 | 100.0 | 100.0 |
| Subject #3 | 18 | 96.6 | 99.7 |  | 100.0 | 98.0 |
| Subject #3 | 19 | 96.1 | 99.9 |  | 100.0 | 98.0 |
| Subject #3 | 20 | 87.9 | 90.5 |  | 97.0 | 91.1 |
| Subject #3 | 21 | 86.2 | 89.9 |  | 96.1 | 90.0 |
| Subject #3 | 22 | 82.4 | 83.0 | 82.8 | 89.0 | 85.0 |
| Subject #3 | 23 | 83.1 | 83.0 | 83.5 | 88.0 | 85.0 |
| Subject #3 | 24 | 79.1 | 79.0 | 80.6 | 82.5 | 81.0 |
| Subject #3 | 25 | 78.8 | 79.1 | 80.0 | 82.4 | 81.0 |
| Subject #3 | 26 | 75.4 | 74.5 | 74.7 | 76.0 | 75.1 |
| Subject #3 | 27 | 73.5 | 74.5 | 76.0 | 78.6 | 74.0 |
| Subject #4 | 1 | 99.5 |  | 97.8 | 100.0 | 100.0 |
| Subject #4 | 2 | 92.1 | 95.7 | 92.0 | 97.0 | 96.0 |
| Subject #4 | 3 | 92.3 | 95.7 | 92.8 | 97.0 | 96.0 |
| Subject #4 | 4 | 93.9 | 95.6 | 93.6 | 97.0 | 96.0 |
| Subject #4 | 5 | 91.3 | 95.1 | 94.0 | 96.0 | 95.2 |
| Subject #4 | 6 | 91.4 | 95.0 | 93.9 | 96.0 | 95.0 |
| Subject #4 | 7 | 91.3 | 95.0 | 93.8 | 96.0 | 95.0 |
| Subject #4 | 8 | 86.8 | 89.4 | 90.2 | 91.0 | 91.0 |
| Subject #4 | 9 | 86.3 | 89.6 | 90.0 | 91.0 | 90.7 |
| Subject #4 | 10 | 84.8 | 88.9 | 89.2 | 90.0 | 88.9 |
| Subject #4 | 11 | 75.0 | 76.9 | 77.6 | 78.0 | 78.0 |
| Subject #4 | 12 | 75.5 | 77.1 | 78.0 | 78.0 | 78.6 |
| Subject #4 | 13 | 75.1 | 77.4 | 78.0 | 78.0 | 78.0 |
| Subject #4 | 14 | 71.8 | 75.6 | 74.5 | 75.0 | 75.0 |
| Subject #4 | 15 | 70.5 | 75.0 | 73.9 | 73.0 | 72.9 |
| Subject #4 | 16 | 69.9 | 74.8 | 73.3 | 72.0 | 72.3 |
| Subject #4 | 17 | 100.0 | 100.0 | 100.0 | 100.0 | 100.0 |
| Subject #4 | 18 | 92.5 | 95.2 | 96.9 | 96.0 | 95.0 |
| Subject #4 | 19 | 93.2 | 95.5 | 97.5 | 97.0 | 95.0 |
| Subject #4 | 20 | 92.2 | 94.7 | 96.0 | 96.0 | 95.0 |
| Subject #4 | 21 | 92.1 | 94.9 | 96.0 | 96.0 | 94.0 |
| Subject #4 | 22 | 89.2 | 91.3 | 92.0 | 92.0 | 92.0 |
| Subject #4 | 23 | 88.5 | 91.7 | 92.1 | 93.0 | 91.0 |
| Subject #4 | 24 | 76.0 | 77.3 | 77.9 | 79.0 | 79.0 |
| Subject #4 | 25 | 75.0 | 77.1 | 77.4 | 78.0 | 78.0 |
| Subject #4 | 26 | 71.7 | 74.5 | 77.1 | 73.0 | 73.6 |
| Subject #4 | 27 | 70.8 | 74.6 | 77.1 | 74.0 | 72.1 |
| Subject #5 | 1 | 97.7 | 100.0 |  | 99.0 | 98.6 |
| Subject #5 | 2 | 96.2 | 97.8 |  | 97.0 | 96.0 |
| Subject #5 | 3 | 95.8 | 98.3 | 98.3 | 97.0 | 96.0 |
| Subject #5 | 4 | 95.5 | 98.4 | 98.7 | 97.0 | 95.9 |
| Subject #5 | 5 | 88.6 | 91.5 |  | 91.0 | 89.9 |
| Subject #5 | 6 | 89.7 | 92.1 |  | 91.5 | 90.7 |
| Subject #5 | 7 | 89.0 | 92.6 |  | 90.4 | 90.0 |
| Subject #5 | 8 | 86.4 | 88.1 | 85.0 | 88.0 | 87.0 |
| Subject #5 | 9 | 85.4 | 88.4 | 85.3 | 88.0 | 87.8 |
| Subject #5 | 10 | 84.6 | 88.1 | 85.2 | 87.0 | 86.3 |
| Subject #5 | 11 | 81.0 | 82.3 |  | 82.0 | 82.0 |
| Subject #5 | 12 | 82.0 | 82.7 |  | 83.0 | 82.0 |
| Subject #5 | 13 | 82.5 | 82.9 |  | 84.0 | 83.0 |
| Subject #5 | 14 | 74.7 | 76.7 |  | 76.0 | 77.0 |
| Subject #5 | 15 | 74.6 | 76.3 |  | 74.3 | 74.6 |
| Subject #5 | 16 | 74.4 | 75.7 |  | 75.0 | 74.9 |
| Subject #5 | 17 | 100.5 |  | 100.0 | 100.0 | 100.0 |
| Subject #5 | 18 | 96.9 |  |  | 98.0 | 97.0 |
| Subject #5 | 19 | 96.8 |  |  | 98.0 | 98.0 |
| Subject #5 | 20 | 93.7 |  |  | 95.0 | 93.0 |
| Subject #5 | 21 | 94.1 |  |  | 95.0 | 94.0 |
| Subject #5 | 22 | 89.4 |  |  | 91.0 | 91.0 |
| Subject #5 | 23 | 90.1 |  |  | 91.0 | 90.0 |
| Subject #5 | 24 | 76.7 |  |  | 77.0 | 82.0 |
| Subject #5 | 25 | 76.3 |  |  | 76.0 | 79.0 |
| Subject #5 | 26 | 73.0 |  |  | 73.0 | 75.0 |
| Subject #5 | 27 | 73.1 |  |  | 73.0 | 73.0 |
| Subject #6 | 1 | 99.5 | 100.0 |  | 99.0 | 100.0 |
| Subject #6 | 2 | 97.3 | 97.5 | 100.0 | 98.0 | 98.0 |
| Subject #6 | 3 | 97.1 | 97.6 | 99.7 | 98.0 | 98.0 |
| Subject #6 | 4 | 97.0 | 98.4 | 100.0 | 98.0 | 98.0 |
| Subject #6 | 5 | 94.0 | 94.3 | 97.3 | 95.0 | 95.0 |
| Subject #6 | 6 | 93.9 | 94.6 | 96.6 | 95.0 | 95.0 |
| Subject #6 | 7 | 94.5 | 95.5 | 94.2 | 94.7 | 96.0 |
| Subject #6 | 8 | 88.7 | 89.3 | 89.8 | 89.0 | 91.0 |
| Subject #6 | 9 | 88.1 | 86.6 | 91.5 | 90.0 | 91.0 |
| Subject #6 | 10 | 87.3 | 87.7 | 90.6 | 88.0 | 89.9 |
| Subject #6 | 11 | 80.4 | 81.6 | 83.5 | 81.0 | 84.0 |
| Subject #6 | 12 | 80.5 | 81.8 | 83.3 | 82.1 | 84.0 |
| Subject #6 | 13 | 80.0 | 81.6 | 82.1 | 81.6 | 84.0 |
| Subject #6 | 14 | 74.4 | 78.7 | 74.5 | 73.3 | 76.5 |
| Subject #6 | 15 | 74.0 | 78.3 | 74.3 | 73.4 | 77.1 |
| Subject #6 | 16 | 71.8 | 77.7 | 73.0 | 72.0 | 74.1 |
| Subject #6 | 17 | 100.2 |  | 100.0 | 100.0 | 100.0 |
| Subject #6 | 18 | 97.8 | 99.7 | 100.0 | 98.0 | 99.0 |
| Subject #6 | 19 | 98.0 | 98.7 | 100.0 | 98.0 | 99.0 |
| Subject #6 | 20 | 94.0 | 96.5 | 98.0 | 94.0 | 96.0 |
| Subject #6 | 21 | 93.5 | 96.8 | 98.0 | 94.0 | 96.0 |
| Subject #6 | 22 | 84.1 | 88.9 | 84.8 | 86.0 | 87.8 |
| Subject #6 | 23 | 83.1 | 87.8 | 84.5 | 85.0 | 87.6 |
| Subject #6 | 24 | 80.2 | 86.0 | 80.9 | 82.0 | 84.0 |
| Subject #6 | 25 | 79.3 | 85.4 | 80.7 | 80.0 | 83.0 |
| Subject #6 | 26 | 73.8 | 80.9 | 76.5 | 75.0 | 77.8 |
| Subject #6 | 27 | 73.1 | 80.8 | 75.2 | 74.9 | 75.6 |
| Subject #7 | 1 | 99.4 |  | 100.0 | 100.0 | 99.8 |
| Subject #7 | 2 | 97.5 | 99.7 | 100.0 | 99.0 | 98.0 |
| Subject #7 | 3 | 97.1 | 99.3 | 100.0 | 99.0 | 98.0 |
| Subject #7 | 4 | 96.9 | 99.3 | 100.0 | 99.0 | 97.4 |
| Subject #7 | 5 | 92.2 | 95.2 |  | 95.3 | 93.6 |
| Subject #7 | 6 | 91.9 | 94.2 | 98.0 | 94.0 | 94.0 |
| Subject #7 | 7 | 91.6 | 93.7 | 96.0 | 94.0 | 93.0 |
| Subject #7 | 8 | 88.4 | 90.0 | 90.0 | 91.0 | 91.0 |
| Subject #7 | 9 | 88.2 | 89.4 | 93.0 | 91.0 | 89.0 |
| Subject #7 | 10 | 87.8 | 89.0 | 93.0 | 90.0 | 89.1 |
| Subject #7 | 11 | 85.3 | 86.3 | 87.0 | 88.9 | 87.0 |
| Subject #7 | 12 | 85.0 | 85.4 | 86.7 | 87.0 | 87.0 |
| Subject #7 | 13 | 84.8 | 85.5 | 86.0 | 88.0 | 86.0 |
| Subject #7 | 14 | 80.3 | 81.8 | 83.0 | 83.5 | 82.6 |
| Subject #7 | 15 | 80.0 | 81.3 | 79.0 | 83.0 | 82.0 |
| Subject #7 | 16 | 79.2 | 80.7 | 81.4 | 83.0 | 81.0 |
| Subject #7 | 17 | 100.3 | 100.0 |  | 100.0 | 100.0 |
| Subject #7 | 18 | 94.3 | 96.6 |  | 96.0 | 94.5 |
| Subject #7 | 19 | 93.5 | 96.7 |  | 96.0 | 95.0 |
| Subject #7 | 20 | 86.0 | 86.8 |  | 88.6 | 88.0 |
| Subject #7 | 21 | 85.4 | 86.5 |  | 88.9 | 87.9 |
| Subject #7 | 22 | 82.1 | 84.0 |  | 85.0 | 85.0 |
| Subject #7 | 23 | 81.3 |  |  |  |  |
| Subject #7 | 24 | 77.9 | 80.5 |  | 81.4 | 81.7 |
| Subject #7 | 25 | 76.3 | 79.0 |  | 79.2 | 80.0 |
| Subject #7 | 26 | 74.9 | 78.2 |  | 78.0 | 79.0 |
| Subject #8 | 1 | 98.5 | 99.3 | 98.6 | 99.0 | 100.0 |
| Subject #8 | 2 | 92.8 | 93.0 | 94.0 | 93.0 | 94.0 |
| Subject #8 | 3 | 93.1 | 93.3 | 93.9 | 93.0 | 94.0 |
| Subject #8 | 4 | 93.2 | 93.6 | 94.0 | 94.0 | 95.0 |
| Subject #8 | 5 | 87.6 | 87.0 | 87.7 | 88.0 | 89.2 |
| Subject #8 | 6 | 87.3 | 87.0 | 87.5 | 87.0 | 89.0 |
| Subject #8 | 7 | 88.3 | 87.3 | 88.2 | 89.0 | 90.0 |
| Subject #8 | 8 | 83.5 | 83.8 | 83.1 | 84.0 | 86.0 |
| Subject #8 | 9 | 83.1 | 83.3 | 83.0 | 84.0 | 85.0 |
| Subject #8 | 10 | 83.6 | 83.3 | 82.7 | 84.0 | 85.8 |
| Subject #8 | 11 | 80.1 | 80.6 | 79.3 | 82.0 | 83.0 |
| Subject #8 | 12 | 80.1 | 80.7 | 79.6 | 81.0 | 83.0 |
| Subject #8 | 13 | 79.5 | 80.8 | 78.8 | 80.9 | 82.0 |
| Subject #8 | 14 | 75.1 | 77.3 | 76.9 | 76.0 | 77.0 |
| Subject #8 | 15 | 73.4 | 76.5 |  | 74.0 | 75.0 |
| Subject #8 | 16 | 72.3 | 76.1 |  | 73.0 | 74.0 |
| Subject #8 | 17 | 100.0 | 100.0 | 98.6 | 100.0 | 100.0 |
| Subject #8 | 18 | 94.2 |  | 94.8 | 95.0 | 96.0 |
| Subject #8 | 19 | 93.8 |  | 94.6 | 95.4 | 95.0 |
| Subject #8 | 20 | 88.0 | 89.7 | 88.6 | 92.0 | 91.0 |
| Subject #8 | 21 | 87.9 | 89.5 | 88.0 | 91.0 | 90.0 |
| Subject #8 | 22 | 85.2 | 86.3 | 84.9 | 89.0 | 88.0 |
| Subject #8 | 23 | 83.6 | 85.7 | 84.3 | 87.4 | 86.0 |
| Subject #8 | 24 | 82.6 | 83.9 | 82.2 | 86.0 | 85.0 |
| Subject #8 | 25 | 82.5 | 84.0 | 82.1 | 87.0 | 85.0 |
| Subject #8 | 26 | 70.3 | 74.1 | 70.0 | 75.0 | 71.0 |
| Subject #8 | 27 | 68.7 | 73.9 | 69.8 | 73.0 | 69.0 |
| Subject #9 | 1 | 98.2 | 99.2 | 98.9 | 99.4 | 99.0 |
| Subject #9 | 2 | 97.9 | 93.3 | 95.1 | 96.0 | 95.0 |
| Subject #9 | 3 | 94.4 | 93.2 | 95.6 | 96.0 | 95.0 |
| Subject #9 | 4 | 94.6 | 93.2 | 95.3 | 96.0 | 95.0 |
| Subject #9 | 5 | 88.9 | 88.0 | 90.4 | 90.0 | 90.0 |
| Subject #9 | 6 | 88.5 | 88.0 | 90.5 | 91.0 | 89.0 |
| Subject #9 | 7 | 88.7 | 87.8 |  | 90.5 | 89.1 |
| Subject #9 | 8 | 86.4 | 86.0 | 85.9 | 88.5 | 87.0 |
| Subject #9 | 9 | 86.0 | 85.8 | 85.4 | 88.0 | 87.0 |
| Subject #9 | 10 | 85.7 | 85.5 | 85.7 | 87.0 | 86.0 |
| Subject #9 | 11 | 76.2 | 74.5 | 74.9 | 76.0 | 73.9 |
| Subject #9 | 12 | 73.5 | 73.5 | 72.9 | 74.0 | 71.0 |
| Subject #9 | 13 | 72.7 | 72.9 | 72.1 | 73.1 | 70.5 |
| Subject #9 | 14 | 71.4 | 72.0 | 70.7 | 73.2 | 69.8 |
| Subject #9 | 15 | 72.4 | 72.5 | 71.6 | 73.0 | 70.0 |
| Subject #9 | 16 | 72.9 | 72.7 | 71.4 | 74.1 | 71.0 |
| Subject #9 | 17 | 100.2 | 100.0 |  | 100.0 | 100.0 |
| Subject #9 | 18 | 93.6 |  | 93.4 | 96.0 | 95.0 |
| Subject #9 | 19 | 93.6 | 93.3 | 93.2 | 96.0 | 95.0 |
| Subject #9 | 20 | 86.3 | 89.2 |  | 89.0 | 88.0 |
| Subject #9 | 21 | 85.8 | 89.3 |  | 88.0 | 87.0 |
| Subject #9 | 22 | 84.9 | 83.7 |  | 87.7 | 86.0 |
| Subject #9 | 23 | 83.9 | 84.0 |  | 86.0 | 85.0 |
| Subject #9 | 24 | 81.5 | 80.4 |  | 84.0 | 82.0 |
| Subject #9 | 25 | 80.7 | 79.5 |  | 83.0 | 82.0 |
| Subject #9 | 26 | 73.5 | 73.2 |  | 74.9 | 72.0 |
| Subject #9 | 27 | 73.2 | 72.7 |  | 74.6 | 71.0 |
| Subject #10 | 1 | 97.0 | 99.5 | 99.5 | 98.0 | 97.7 |
| Subject #10 | 2 | 90.2 | 91.8 | 91.2 | 93.0 | 91.0 |
| Subject #10 | 3 | 90.0 | 91.6 | 91.7 | 92.4 | 91.0 |
| Subject #10 | 4 | 92.6 | 92.9 | 93.2 | 94.0 | 93.0 |
| Subject #10 | 5 | 86.8 | 89.5 | 88.2 | 90.1 | 88.2 |
| Subject #10 | 6 | 88.0 | 89.1 | 88.7 | 91.0 | 90.0 |
| Subject #10 | 7 | 86.3 | 89.7 | 88.3 | 90.4 | 90.0 |
| Subject #10 | 8 | 87.2 | 87.5 | 88.0 | 90.0 | 88.1 |
| Subject #10 | 9 | 83.9 | 86.7 | 86.7 | 89.0 | 86.0 |
| Subject #10 | 10 | 84.3 | 86.3 | 87.5 | 89.0 | 86.8 |
| Subject #10 | 11 | 81.9 | 82.7 | 83.9 | 87.0 | 84.2 |
| Subject #10 | 12 | 81.0 | 82.1 | 82.9 | 86.0 | 83.4 |
| Subject #10 | 13 | 78.5 | 81.3 | 81.6 | 83.6 | 80.9 |
| Subject #10 | 14 | 73.9 |  | 77.4 | 79.5 | 75.6 |
| Subject #10 | 15 | 74.8 |  |  | 80.3 | 77.0 |
| Subject #10 | 16 | 72.7 | 75.5 | 74.9 | 79.0 | 75.0 |
| Subject #10 | 17 | 100.2 | 100.0 | 100.0 | 100.0 | 100.0 |
| Subject #10 | 18 | 95.2 | 97.1 | 97.1 | 98.0 | 96.7 |
| Subject #10 | 19 | 94.3 | 95.4 | 94.5 | 95.4 | 94.9 |
| Subject #10 | 20 | 84.3 | 84.4 | 84.9 | 88.9 | 86.2 |
| Subject #10 | 21 | 81.7 | 84.2 | 84.1 | 83.1 | 82.8 |
| Subject #10 | 22 | 78.2 | 79.4 | 80.0 | 83.0 | 80.2 |
| Subject #10 | 23 | 76.7 | 79.4 | 79.8 | 81.5 | 79.0 |
| Subject #10 | 24 | 76.4 | 78.5 | 78.5 | 82.0 | 79.0 |
| Subject #10 | 25 | 76.0 | 78.2 | 78.3 | 81.7 | 79.0 |
| Subject #10 | 26 | 73.1 | 76.5 | 76.0 | 78.0 | 75.0 |
| Subject #10 | 27 | 72.4 | 76.2 | 74.9 | 77.6 | 74.0 |
| Subject #11 | 1 | 98.8 | 100.0 | 95.0 | 98.0 | 98.0 |
| Subject #11 | 2 | 95.7 | 94.1 | 90.1 | 95.0 | 95.0 |
| Subject #11 | 3 | 95.8 | 94.2 | 90.3 | 95.0 | 95.0 |
| Subject #11 | 4 | 95.9 | 94.2 | 90.8 | 95.0 | 95.0 |
| Subject #11 | 5 | 91.8 | 91.0 | 88.6 | 91.0 | 91.0 |
| Subject #11 | 6 | 91.6 | 91.0 | 88.5 | 91.0 | 91.0 |
| Subject #11 | 7 | 92.1 | 91.0 | 88.5 | 91.0 | 91.0 |
| Subject #11 | 8 | 88.2 | 87.0 | 86.2 | 88.0 | 87.4 |
| Subject #11 | 9 | 87.7 | 87.0 | 86.3 | 87.6 | 87.0 |
| Subject #11 | 10 | 87.6 | 87.2 | 86.0 | 88.0 | 87.0 |
| Subject #11 | 11 | 78.5 | 76.7 | 79.1 | 79.0 | 77.0 |
| Subject #11 | 12 | 77.6 | 75.7 | 78.3 | 77.0 | 75.0 |
| Subject #11 | 13 | 75.2 | 74.5 | 77.7 | 75.0 | 72.7 |
| Subject #11 | 14 | 75.0 | 73.7 | 76.6 | 75.0 | 72.2 |
| Subject #11 | 15 | 74.8 | 73.6 | 76.9 | 74.0 | 71.7 |
| Subject #11 | 16 | 73.8 | 72.9 | 76.9 | 72.7 | 70.0 |
| Subject #11 | 17 | 100.3 | 100.0 | 100.0 | 100.0 | 100.0 |
| Subject #11 | 18 | 94.8 | 94.6 | 94.5 | 95.0 | 94.7 |
| Subject #11 | 19 | 94.8 | 94.3 | 94.0 | 94.0 | 94.0 |
| Subject #11 | 20 | 88.8 | 88.1 | 87.5 | 88.0 | 88.0 |
| Subject #11 | 21 | 88.8 | 88.0 | 87.2 | 88.0 | 88.0 |
| Subject #11 | 22 | 84.0 | 82.3 | 83.4 | 84.0 | 83.0 |
| Subject #11 | 23 | 83.9 | 82.0 | 83.3 | 83.9 | 83.0 |
| Subject #11 | 24 | 81.4 | 80.0 | 81.6 | 81.0 | 81.0 |
| Subject #11 | 25 | 82.0 | 80.3 | 82.0 | 82.0 | 81.0 |
| Subject #11 | 26 | 73.8 | 72.8 | 76.0 | 73.0 | 71.0 |
| Subject #11 | 27 | 72.2 | 72.6 | 76.0 | 71.1 | 69.0 |
| Subject #12 | 1 | 97.9 | 100.0 | 97.2 | 98.0 | 97.0 |
| Subject #12 | 2 | 92.3 | 92.6 | 92.9 | 98.0 | 92.2 |
| Subject #12 | 3 | 92.2 | 91.8 | 92.2 | 97.5 | 92.0 |
| Subject #12 | 4 | 91.6 | 91.4 | 91.7 | 93.4 | 92.0 |
| Subject #12 | 5 | 88.0 | 87.7 | 88.1 | 90.0 | 88.6 |
| Subject #12 | 6 | 88.2 | 87.8 | 88.3 | 90.0 | 89.0 |
| Subject #12 | 7 | 88.5 | 88.3 | 88.9 | 90.5 | 89.0 |
| Subject #12 | 8 | 85.4 | 84.9 | 85.4 | 87.0 | 86.0 |
| Subject #12 | 9 | 84.9 | 84.4 | 85.0 | 87.0 | 85.0 |
| Subject #12 | 10 | 84.8 | 84.6 | 85.6 | 87.5 | 86.0 |
| Subject #12 | 11 | 78.3 | 78.8 | 76.7 | 82.2 | 80.3 |
| Subject #12 | 12 | 78.0 | 77.8 | 78.2 | 81.3 | 79.5 |
| Subject #12 | 13 | 78.3 | 77.4 | 78.7 | 81.0 | 80.0 |
| Subject #12 | 14 | 72.6 | 72.2 | 73.3 | 76.4 | 74.7 |
| Subject #12 | 15 | 70.6 | 71.8 | 72.3 | 75.0 | 72.0 |
| Subject #12 | 16 | 69.9 | 71.5 | 72.5 | 74.0 | 71.0 |
| Subject #12 | 17 | 100.5 | 100.0 | 96.7 | 100.0 | 100.0 |
| Subject #12 | 18 | 95.2 | 94.5 |  | 95.5 | 95.0 |
| Subject #12 | 19 | 95.0 | 95.0 | 94.9 | 96.0 | 95.0 |
| Subject #12 | 20 | 86.6 | 87.2 | 84.5 | 89.0 | 87.0 |
| Subject #12 | 21 | 85.3 | 86.8 | 84.7 | 87.0 | 85.8 |
| Subject #12 | 22 | 81.0 | 80.6 | 80.7 | 84.0 | 82.0 |
| Subject #12 | 23 | 80.5 | 80.0 | 80.0 | 83.0 | 82.0 |
| Subject #12 | 24 | 75.1 | 74.7 | 74.5 | 79.0 | 77.0 |
| Subject #12 | 25 | 75.1 | 74.6 | 74.8 | 78.0 | 77.0 |
| Subject #12 | 26 | 69.9 | 70.9 | 69.5 | 74.0 | 71.2 |
| Subject #12 | 27 | 68.0 | 70.7 | 69.7 | 72.0 | 70.0 |
